# Deciphering the Molecular Landscape of Squamous Cell Carcinoma of the Anal Canal: From Biology to Precision Oncology

**DOI:** 10.3390/cancers18162602

**Published:** 2026-08-12

**Authors:** Matilde Callegarin, Valentina Angerilli, Jessica Gasparello, Francesca Bergamo, Rodrigo Humberto Giron Cuestas, Paola Parente, Sara Lonardi, Matteo Fassan

**Affiliations:** 1 Department of Medicine-DIMED, University of Padua, 35100 Padova, Italy; matilde.callegarin@studenti.unipd.it (M.C.); jessica.gasparello@unipd.it (J.G.); 2 Surgical Pathology Unit, ULSS2 Marca Trevigiana, 31100 Treviso, Italy; valentina.angerilli@aulss2.veneto.it; 3 Department of Pathology, Radboud Institute for Molecular Life Sciences, Radboud University Medical Centre, 6525 GA Nijmegen, The Netherlands; 4 Veneto Institute of Oncology IOV-IRCCS, 35128 Padua, Italy; francesca.bergamo@iov.veneto.it (F.B.); sara.lonardi@iov.veneto.it (S.L.); 5 Department of Anatomical Pathology and Cytopathology, University Hospital “Dr. José Eleuterio González”, Autonomous University of Nuevo León (UANL), Monterrey 64000, Nuevo Leon, Mexico; rodcuestas1@gmail.com; 6 Pathology Unit, Azienda Ospedaliero-Universitaria Policlinico Foggia, 71122 Foggia, Italy; paolaparente77@gmail.com

**Keywords:** squamous cell carcinoma, anal carcinoma, biomarkers, predictive biomarkers

## Abstract

Squamous cell carcinoma of the anal canal (SCAC) is a rare malignancy whose incidence has been steadily increasing worldwide. Persistent infection with high-risk human papillomavirus (HPV), particularly HPV16 and HPV18 genotypes, is the main etiological factor and plays a central role in tumor development. While combined chemoradiotherapy remains the standard treatment for localized disease and achieves high rates of tumor control, a considerable proportion of patients experience recurrence or present with advanced disease. For the latter, therapeutic options remain limited. Over the last decade, advances in genomic profiling have significantly expanded our understanding of SCAC biology. Recurrent alterations affecting the PI3K/AKT/mTOR pathway, especially *PIK3CA* mutations, have emerged as the most common molecular events, particularly in HPV-positive tumors. Additional alterations involve receptor tyrosine kinase signaling, chromatin remodeling genes, DNA damage response pathways, and components of the MAPK cascade. Moreover, HPV-positive and HPV-negative tumors display distinct molecular features with important prognostic implications. Immunotherapy has recently become an important component of treatment for advanced SCAC, although reliable predictive biomarkers are still lacking. This review summarizes the current evidence on the molecular landscape of SCAC, discusses emerging prognostic and predictive biomarkers, and highlights potential opportunities for the development of more personalized therapeutic strategies.

## Simple Summary

Squamous cell carcinoma of the anal canal (SCAC) is a rare malignancy whose incidence has been increasing worldwide. The main etiological factor is HPV infection, particularly in association with HIV infection and other immunosuppressive conditions. The majority of patients present with localized disease and are treated with chemoradiotherapy, but a significant percentage experience relapses, and approximately 10% present with advanced disease. In the advanced setting, the main therapeutic strategies are systemic chemotherapy and, more recently, immunotherapy, but the prognosis remains poor. In this context, the identification of novel personalized treatments and reliable predictive biomarkers is crucial.

## 1. Introduction

Anal cancer is a rare malignancy, accounting for less than 3% of gastrointestinal cancers, but its incidence has been increasing worldwide [1,2]. Persistent infection with HPV represents the main risk factor and is detected in up to 90% of cases, most frequently involving the high-risk genotype HPV16, followed by HPV18 [3]. Additional factors are involved in the subsequent stages of carcinogenesis, the main include HIV infection, other sexually transmitted diseases, acquired immunosuppression, and smoking [4,5,6,7].

The most frequent histological subtype is squamous cell carcinoma of the anal canal (SCAC). SCAC is a morphologically heterogenous disease, comprising cases with basaloid, adenoid cystic or cylindroma-like and urothelial carcinoma-like appearance [8,9,10]. Since the identification of these histological patterns has no impact on prognosis or clinical management and it is burdened by a high level of interobserver variability, histological subtyping is not recommended by the 6th edition of the World Health Organization (WHO) classification [11]. The only exception is represented by the verrucous carcinoma subtype, a rare and well-differentiated variant not associated with HPV infection, which is locally destructive but does not metastasize [11].

For patients with localized disease, the standard of care is radiotherapy in combination with 5-fluorouracil (5-FU) and mitomycin C (MMC), which allows complete tumor regression in 80–90% of cases [2]. However, a significant proportion of patients experience locoregional recurrence, and approximately 10% of cases present with metastatic disease [2]. Chemotherapy, and more recently immunotherapy, have demonstrated some effectiveness in advanced disease, but responses are often modest, with a 5-year survival rate of 30% [2].

In recent years, advances in genomic profiling have substantially improved the understanding of the molecular landscape of SCAC. Alterations in *PIK3CA* have been frequently reported in HPV-positive tumors, along with mutations in other genes involved in the PIK3CA/AKT/mTOR signaling pathway. On the contrary, *CDKN2A* and *TP53* alterations have been frequently described in HPV-negative tumors. Furthermore, recurrent alterations in genes involved in several other signaling pathways have been reported (Table 1) [12].

Despite these advances, the molecular characterization of SCAC remains incomplete. Most of the available studies have focused on small cohorts and are characterized by substantial heterogeneity, including differences in patient populations, sequencing platforms, and variant calling thresholds. Large-scale initiatives such as The Cancer Genome Atlas (TCGA) have not comprehensively addressed this disease. As a result, clinically validated biomarkers and molecular classifications are still lacking.

In this review, we provide a comprehensive overview of the current knowledge on the molecular landscape of SCAC, highlighting emerging prognostic and predictive biomarkers. Indeed a deeper understanding of the molecular characteristics of SCAC is essential to develop more effective and personalized therapeutic strategies.

## 2. Molecular Characterization and Potentially Actionable Alterations in SCAC

### 2.1. PI3K/AKT/mTOR Pathway

The PI3K/AKT/mTOR pathway represents the most frequently altered signaling cascade in SCAC.

**Figure 1:**
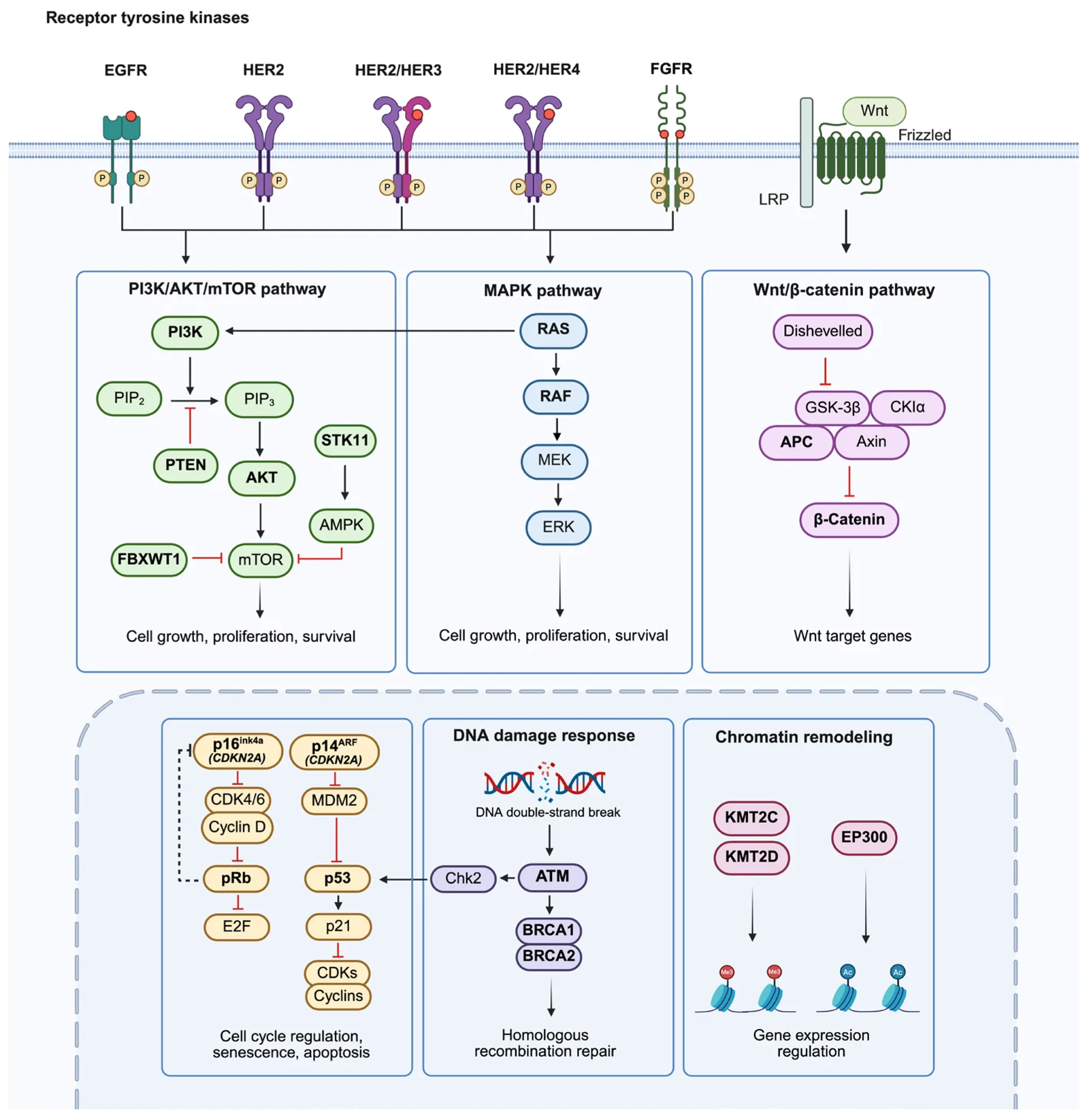
Overview of the main signaling pathways altered in SCAC.

Several studies consistently reported the *PIK3CA* gene as the most frequently altered in SCAC, with mutations and/or amplifications occurring in approximately 30% of cases [10,13,14,15,16,17,18,19,20,21,22]. *PIK3CA* encodes the p110α catalytic subunit of phosphatidylinositol-3-kinase (PI3K), which is normally activated by upstream receptor tyrosine kinases such as EGFR and FGFR [23]. Once activated, PI3K phosphorylates phosphatidylinositol-4,5-bisphosphate (PIP2) to form phosphatidylinositol-3,4,5-trisphosphate (PIP3), which in turn recruits the serine/threonine kinase AKT. Activated AKT subsequently phosphorylates multiple downstream effectors, including mTOR, thus promoting tumor cell survival, proliferation, and the remodeling of cellular metabolism (Figure 1) [23].

The most frequent *PIK3CA* alterations reported in SCAC are p.E545K and p.E542K point mutations occurring in the helical domain [13], that result in a decrease in p110α suppression, mediated by the p85 regulatory subunit [24]. Less frequent alterations include *PIK3CA* amplifications, which account for approximately 20% of cases, and mutations involving the kinase domain, such as p.H1047R, as well as other rarer variants [13]. The prognostic significance of *PIK3CA* alterations is still unclear. While some studies have identified these factors as negative prognostic indicators in patients undergoing abdominoperineal resection for local recurrence [21,25], others found no association with response to chemotherapy in treatment-naïve advanced tumors [26].

Dysregulation of the PI3K/AKT/mTOR signaling is not limited to PIK3CA. Additional alterations have been reported in several other genes, part of the same molecular mechanisms, including *PTEN*, *AKT1*, *STK11*, and *FBXW7*, with an overall prevalence of alterations in this pathway of approximately 60% [20,22].

PTEN is a tumor suppressor that mediates the inhibition of PI3K signaling by dephosphorylating PIP3 and converting it back to PIP2 [24]. *PTEN* mutations have been found in 13% of SCAC cases [10,13,14,15,16,17,20,22]. Notably, concurrent *PTEN* gene expression loss and *PIK3CA*-mutation have been frequently described in HPV-positive squamous cell carcinomas, including SCAC [27], and could promote resistance to treatments with PI3K inhibitors [28].

FBXW7 is another tumor suppressor which mediates the ubiquitination and subsequent degradation of several molecules, including the master regulatory protein kinase mTOR [29]. Mutations in the *FBXW7* gene have been reported in 12% of SCAC cases [10,13,14,15,16,17,18,20,21,22].

The high frequency of alterations in the PI3K/AKT/mTOR signaling pathway provides a strong rationale for the investigation of targeted therapies directed against its components. However, the level of evidence regarding the efficacy of this approach in SCAC is still very limited. PI3K inhibitors such as alpelisib are currently approved by the FDA and EMA for the treatment of locally advanced or metastatic breast cancer presenting *PIK3CA* mutations. Studies on other HPV-related squamous cell carcinomas, such as cervical cancer and head and neck squamous cell carcinoma (HNSCC), have reported improved survival in patients with *PIK3CA* or *PTEN* alterations treated with therapies targeting the PI3K/AKT/mTOR signaling pathway [20]. In addition, a recent study that molecularly characterized the largest cohort of SCAC patients to date (1844 patients) also reported stable disease with excellent tolerability in a patient with metastatic SCAC enrolled in a PI3K inhibitor trial after the identification of a *PIK3CA* mutation in circulating tumor DNA (ctDNA) [13]. Nevertheless, larger prospective studies are needed to validate PI3K/AKT/mTOR pathway alterations as actionable targets in SCAC.

### 2.2. Receptor Tyrosine Kinases

#### 2.2.1. EGFR

Epidermal growth factor receptor (EGFR) is a transmembrane receptor tyrosine kinase part of the ErbB family that, upon ligand binding and dimerization, activates intracellular signaling cascades including the PI3K/AKT/mTOR and the RAS/RAF/MEK/ERK pathways, thereby promoting proliferation, survival, migration, and resistance to apoptosis (Figure 1) [30]. Studies based on immunohistochemistry (IHC) have demonstrated EGFR expression in the great majority of SCAC cases, with reported IHC positivity rates of 90–98% [16,22]. However, this high prevalence of EGFR protein expression is not associated with a similar frequency of *EGFR* molecular alterations, which overall have been reported in only 2% of cases [10,13,15,16,19,20,22]. Recently, a large comprehensive genomic profiling study identified *EGFR* alterations by NGS in 2.5% of cases [13]. The most frequent genetic alterations comprise amplifications, followed by missense mutations and rare rearrangements [13].

The high rate of EGFR expression, together with the low frequency of downstream mutations in *KRAS*, *NRAS*, and *BRAF*, provided the rationale for testing EGFR-targeted therapies in SCAC. Early small studies suggested a possible activity of cetuximab in *KRAS* wild-type metastatic disease [31]. However, prospective trials combining EGFR inhibitors with chemoradiotherapy (CRT) in stage I-III SCAC have not yielded satisfactory results. In the UNICANCER ACCORD 16, ECOG E3205, AMC045, and FFCD 0904 trials, the addition of cetuximab or panitumumab to CRT was associated with substantial toxicity, including high rates of grade 4 adverse events and treatment-related deaths, without consistent improvement in oncologic outcomes [32,33,34,35]. On the other hand, in the phase II CARACAS trial, the combination of cetuximab plus the anti-PD-L1 antibody avelumab in previously treated, locally advanced or metastatic SCAC was associated with a higher overall response rate (ORR) compared to avelumab alone, with an acceptable safety profile [36]. This result suggests a promising activity of dual EGFR and PD-L1 blockade in advanced SCAC [36].

Overall, the current evidence does not support routine EGFR-targeted therapy or *EGFR* testing based only on IHC expression. *EGFR* amplification, although rare, may define a small subgroup of tumors with stronger biological dependence on EGFR signaling and deserves further investigation as a potential predictive biomarker for anti-EGFR therapy, especially in the advanced setting.

#### 2.2.2. HER2

HER2, also known as ErbB2, is a receptor tyrosine kinase belonging to the ErbB family, which also includes EGFR/ErbB1, ErbB3 and ErbB4 [37,38]. HER2 alterations in SCAC appear to be exceedingly rare, with reported frequencies of *ERBB2* molecular alterations of 2% [13,16,20,22], and of HER2 overexpression by IHC of less than 1% [16]. Nonetheless, HER2-targeted therapies have demonstrated clinical efficacy and are approved in several malignancies, including breast, gastric/gastro-esophageal junction (G/GEJ) and colorectal cancers (CRC) [39,40,41]. Recent studies have also shown promising activity across multiple other solid tumors [42,43]. Given the rarity of HER2 alterations in SCAC, the inclusion of patients with *ERBB2* amplification and/or HER2 overexpression in molecularly selected and tumor-agnostic clinical trials may help clarify the relevance of HER2-targeted therapies in this disease.

#### 2.2.3. FGFR

Fibroblast growth factor receptors (FGFR) are a family of receptor tyrosine kinases comprising FGFR1, FGFR2, FGFR3 and FGFR4, which are involved in cell survival, proliferation, angiogenesis and differentiation (Figure 1) [44]. Their aberrant activation through single-nucleotide mutations, amplifications or fusions can contribute to oncogenic transformation [44].

Available data indicate a low prevalence of FGFRs alterations in SCAC, with *FGFR3* alterations reported in 3% of cases [10,13,16,17,20] and *FGFR1* and *FGFR2* alterations in 1% [13,16,19,20,22]. Pan-FGFR inhibitors are currently approved for the treatment of cholangiocarcinomas and urothelial cancers [45,46]. In SCAC, Miranda et al. reported an excellent response to the FGFR1-3 inhibitor pemigatinib in a patient with recurrent, metastatic disease and FGFR1 overexpression [47]. Another case report demonstrated stable disease and a progression-free survival (PFS) of 4 months in a patient with a *FGFR2* point-mutation (p.K659N) treated with a FGFR-inhibitor, with primary resistance to the treatment presumably related to the presence of concurrent *NF1* and *PTEN* alterations [13].

Overall, *FGFR* genes alterations in SCAC appear to be rare but potentially actionable, warranting further investigation in molecularly selected patients.

### 2.3. MAPK Pathway

RAS and BRAF act downstream of EGFR as part of the RAS-RAF-MEK-ERK cascade, a key pathway that regulates cellular proliferation, differentiation and apoptosis (Figure 1) [48]. Gain-of-function mutations in *RAS* or *BRAF* can lead to constitutive activation of the MAPK pathway, bypassing upstream receptor regulation and promoting oncogenesis [48].

Several studies have shown that *RAS* or *BRAF* mutations are infrequent in SCAC, a finding that is also consistent with the molecular profile of other HPV-associated squamous cell carcinomas [19,49]. More in detail, *KRAS* alterations have been reported in 2% of cases [10,13,14,16,19,20,21,22], while *NRAS* and *HRAS* alterations have been found in less than 1% of patients [13,15,16,19,20,21,22]. In the largest study on SCAC to date, *KRAS* alterations were found in 2.4% of patients, including missense mutations (92.7%) and, less frequently, amplifications (7.3%) [13]. Three patients (7.3%) harbored a c.G12C point-mutation, which can be targeted by KRAS-G12C inhibitors such as sotorasib, currently approved by the FDA for NSCLC and CRC [50]. In addition, Pan-KRAS inhibitors are currently under investigation [51].

*BRAF* alterations have been reported in 1% of patients [10,13,14,16,19,21,22], including the *BRAF* p.V600E mutation [13]. The latter can be targeted by specific inhibitors such as encorafenib, currently approved for melanoma, CRC and NSCLC.

Although rare, *RAS* and *BRAF* mutations represent potential therapeutic targets, and the inclusion of patients with SCAC in clinical trials with agents directed against these molecules could allow the identification of novel effective treatments.

### 2.4. Histone Modification and Chromatin Remodeling

Alterations in genes involved in chromatin remodeling and histone modification appear to be frequent in SCAC. Following *PIK3CA*, the genes encoding the methyltransferases KMT2D (or MLL2/MLL4) and KMT2C (or MLL3) are among the most frequently altered, with a prevalence of 18% and 14%, respectively [10,13,14,16,17,18,19,20]. KMT2D and KMT2C are histone H3 lysine 4 (H3K4) methyltransferases involved in the mono-methylation of lysine residues at enhancer regions, promoting genome accessibility and consequently gene transcription [52]. Loss-of-function mutations in these genes are frequently observed across several cancer types and have been implicated in multiple oncogenic processes, including metabolic dysregulation and evasion of growth-suppression cellular mechanisms [53]. Although their relevance in SCAC is not completely defined, emerging evidence from other solid tumors suggests that *KMT2D* and *KMT2C* loss-of-function mutations may also be associated with increased tumor-infiltrating lymphocytes, elevated PD-L1 expression and microsatellite instability, therefore correlating with the response to immune checkpoint inhibitors [53,54,55].

In addition, mutations in EP300, a histone acetyltransferase involved in the transcriptional regulation of genes involved in proliferation and differentiation [56], have also been reported in 6% of cases [10,13,14,16,19,20].

### 2.5. DNA Damage Response

Alterations in genes involved in DNA damage response have been described in a subset of SCAC cases. The most frequent include *BRCA1* and *BRCA2* mutations, found in 3% of cases each [13,16,18,20,22], and *ATM* mutations, reported in 4% of cases [15,16,18,20,22]. These genes encode key components of the homologous recombination repair (HRR) mechanism, essential for the repair of DNA double-strand breaks [57]. Alterations in these proteins result in a phenotype known as homologous recombination deficiency (HRD), which has been associated with sensitivity to poly(ADP-ribose) polymerase (PARP) inhibitors [57]. PARP is normally involved in the repair of DNA single-strand breaks, and its inhibition leads to the development of DNA double-strand breaks [58,59]. In neoplastic cells with HRD, this damage cannot be properly repaired, and the intervention of error-prone DNA repair mechanisms ultimately results in genomic instability and cell death [58,59]. PARP inhibitors are currently approved for the treatment of HRD breast, ovarian, prostate, and pancreatic cancers [60]. This strategy was initially adopted in patients with *BRCA1* or *BRCA2* mutations, but has later been explored in tumors with alterations in other genes involved in HRR, such as *RAD51C* and *RAD51D* [60,61,62]. A recent study reported a PFS of six months in a patient with SCAC harboring a *RAD51C* mutation treated with a PARP inhibitor [13], but additional studies are required to evaluate the clinical relevance of PARP inhibitors in this setting.

### 2.6. Wnt/β-Catenin Pathway

The Wnt/β-catenin signaling pathway is frequently dysregulated in cancer. In normal cells, in the absence of Wnt ligands, free β-catenin is targeted by a destruction complex composed of adenomatous polyposis coli (APC), Axin, casein kinase 1 (CK1) and glycogen synthase kinase 3 (GSK3) [63]. The binding of Wnt ligands to the receptors Frizzled and LRP5/6 leads to the inhibition of the destruction complex, thereby allowing the translocation of β-catenin to the nucleus and its interaction with the transcription factor TCF/LEF, ultimately resulting in the transcription of Wnt target genes (Figure 1) [63]. These are involved in cell proliferation, survival and differentiation, and are essential for embryogenesis and stem cell maintenance [63]. Alterations in this pathway are involved in tumor initiation and progression [64].

In contrast to CRC, in which biallelic inactivation of *APC* is found in approximately 80% of cases and Wnt/β-catenin pathway is altered in more than 90% of tumors [65], *APC* mutations have been reported in only 3% of SCAC cases [10,16,20,22]. Similarly, alterations in the *CTNNB1* gene, encoding β-catenin, have been described in only 2% of cases [10,16,20,22]. Mutations in *FAT1*, which encodes a transmembrane protocadherin, have been reported in 9% of cases [10,17,18]. Loss of function mutations in *FAT1* have been associated with activation of Wnt signaling and tumorigenesis, and are frequently reported in other tumors, including HNSCC [66]. Although the relevance of *FAT1* alterations in SCAC has not yet been established, in patients with HNSCC they have been associated with HPV-negative status [67].

Therapeutic agents targeting the Wnt/β-catenin signaling pathway are not currently approved and adopted in clinical practice, but several strategies are under investigation [63,68].

## 3. Differences Between HPV-Positive and HPV-Negative SCAC

HPV infection is well established as the main risk factor for SCAC, found in approximately 90% of cases [3]. The subtype most frequently associated with SCAC is HPV16 (accounting for about 70% of all cases), followed by HPV18 [3]. HPV infects epithelial cells and promotes neoplastic transformation mainly through the expression of the E6 and E7 oncoproteins [69]. More in detail, E6 promotes the degradation of the tumor suppressor protein p53, thus limiting the ability of the cell to induce growth arrest and apoptosis, whereas E7 promotes the degradation of pRb, leading to the release of E2F transcription factors and allowing the progression into the S phase of the cell cycle [70,71]. pRb also negatively regulates p16 expression through a feedback mechanism; as a result, HPV-positive tumors frequently exhibit p16 overexpression [72,73]. Since diffuse p16 expression evaluated by IHC is sensitive but not entirely specific for the identification of HPV-driven tumors, in situ hybridization or molecular testing may be required to confirm HPV status [72].

**Figure 2:**
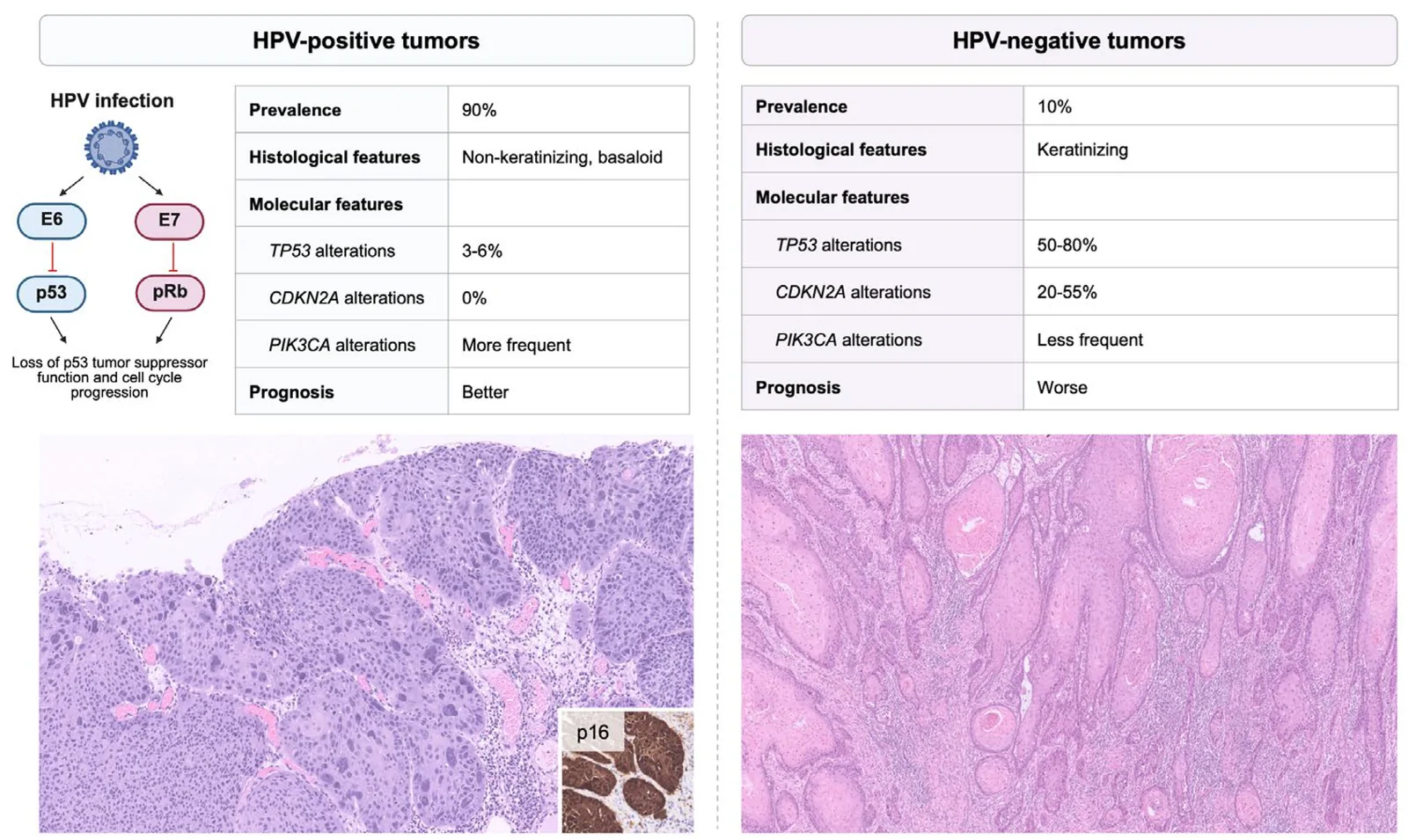
Differences in clinical, molecular and histological features between HPV-positive and HPV-negative SCAC.

Histologically, HPV-negative SCAC is typically associated with a keratinizing morphology, while HPV-associated tumors are more frequently non-keratinizing, often with a basaloid appearance (Figure 2) [11,74]. The verrucous carcinoma subtype, which is characterized by a pushing growth pattern and lacks severe cytological atypia, is not associated with HPV infection [11].

HPV-positive and HPV-negative tumors are also characterized by distinct molecular profiles (Figure 2). Loss-of-function mutations in the tumor suppressor gene *TP53* are significantly more frequent in HPV-negative tumors than in HPV-positive ones (50–80% vs. 3–6%) [12,20,72,75]. Similarly, alterations in the *CDKN2A* gene have been reported in 20–55% of HPV-negative tumors and are generally absent in HPV-positive ones [12,20,72]. *CDKN2A* encodes p16^INK4a^ and p14^ARF^, two proteins involved in cell cycle regulation that promote the activation of pRb and p53, respectively (Figure 1) [76]. This discrepancy in mutations rates is consistent with the expression of the E6 and E7 oncoproteins in HPV-positive SCAC, which results in the functional inactivation of p53 and pRb signaling pathways without requiring additional *TP53* or *CDKN2A* somatic mutations. By contrast, mutations in *PIK3CA* are more frequent in HPV-positive SCAC, suggesting a prominent role of PI3K/AKT/mTOR pathway activation in HPV-driven tumors [12,13,20,72]. More in detail, a large study reported *PIK3CA* alterations in 39.0% of HPV16-positive cases, while they were less frequently reported in HPV-negative cases (19.3%) and in other less common HPV genotypes [13]. A similar pattern was observed for *PTEN* (14.5% in HPV16-positive versus 6.9% in HPV-negative cases), *FBXW7* (13.8% versus 5.6%), and *KMT2D* alterations (20.8% versus 8.6%) [13]. Conversely, *TERT* alterations were substantially more frequent in HPV-negative than in HPV16-positive cases (34.8% versus 1.9%, respectively) [13].

The distinction between HPV-positive and HPV-negative neoplasms also has potential clinical implications, and ESMO guidelines currently recommend p16/HPV assessment in the diagnostic work-up of SCAC [2]. HPV-positive tumors are generally associated with more favorable outcomes after CRT and improved overall survival (OS) [72,77,78]. Furthermore, *TP53* mutations and aberrant p53 IHC have been associated with worse outcomes among HPV-negative tumors [72]. These findings support the clinical relevance of classifying SCAC according to HPV status, and suggest that the combined assessment of p53 and p16 expression by IHC could improve prognostic stratification [72].

Given their more favorable prognosis, treatment de-escalation strategies could potentially be considered for patients with HPV-positive SCAC. Preliminary findings suggested that de-escalation of CRT for HPV-positive tumors may reduce acute toxicities while achieving similar outcomes compared to standard CRT [79]. However, additional prospective studies are needed to determine whether HPV positivity can reliably guide treatment de-escalation or, conversely, whether HPV-negative tumors may benefit from treatment intensification.

## 4. Immunotherapy and Associated Predictive Biomarkers

The strong association between SCAC and HPV infection also provides a biological rationale for immunotherapy. HPV oncoproteins E6 and E7 promote immune escape through the PD-1/PD-L1 axis and through modulation of the immune microenvironment, and HPV-driven tumors are usually associated with a high level of immunogenicity [80]. Therefore, immune checkpoint inhibitors (ICIs) represent a possible therapeutic strategy aimed at restoring antitumor immunity [80].

In localized and locally advanced disease, immunotherapy is currently being investigated in combination with standard CRT, either as induction, concurrent, and/or consolidative treatment.

The phase II INTERACT-ION trial evaluated a strategy based on induction therapy with modified docetaxel, cisplatin, and fluorouracil (mDCF) plus the anti-PD-1 antibody ezabenlimab followed by response-adapted CRT in patients with locally advanced SCAC [81]. Patients with a good response received involved-node chemoradiotherapy (INRT) and subsequent ezabenlimab maintenance therapy, whereas poor responders received standard CRT [81]. Ezabenlimab plus mDCF induction provided high pathological complete or near-complete response rates, allowing subsequent treatment with INRT in 75% of patients, and the study met its primary endpoint, demonstrating a clinical complete response in 78% of patients at 40 weeks, with a manageable safety profile [81].

Other ongoing studies are evaluating the combination of CRT and ICIs in different treatment phases. The phase II RADIANCE trial is assessing the addition of the anti-PD-L1 antibody durvalumab to standard CRT in locally advanced SCAC, with 3-year disease-free survival as the primary endpoint [82]. The phase III EA2165 trial (NCT03233711) is evaluating nivolumab after CRT in patients with high-risk stage II-IIIB disease. Additional trials are investigating the combination of CRT with other ICIs, including pembrolizumab (NCT04046133), nivolumab (NCT04929028), toripalimab (NCT05060471) and atezolizumab plus tiragolumab (NCT05661188).

In advanced or metastatic SCAC, PD-1/PD-L1 blockade has shown modest but durable activity in patients with previously treated disease [80].

Nivolumab monotherapy was evaluated in the phase II NCI9673 (Part A) trial in 37 patients with unresectable metastatic disease, and was associated with an ORR of 24%, including complete and partial responses, a median PFS of 4.1 months, and a median OS of 11.5 months [83]. The randomized NCI9673 (Part B) trial subsequently compared nivolumab alone with nivolumab plus the anti-CTLA-4 antibody ipilimumab. However, the addition of ipilimumab did not statistically improve ORR, PFS, or OS, and was associated with increased toxicity [84].

Pembrolizumab demonstrated a clinical benefit in the multicohort phase Ib KEYNOTE-028 trial, which enrolled 24 patients with PD-L1-positive (combined positive score, CPS ≥ 1) advanced SCAC and reported an ORR of 17% [85], and in the phase II KEYNOTE-158, which included 112 patients with SCAC and reported an ORR of 11%, a median PFS of 2 months and a median OS of 11.9 months [86].

The phase II CARACAS trial, which evaluated avelumab alone or in combination with cetuximab, reported an ORR of 10%, a PFS of 2.0 months and a median OS of 13.9 months in patients treated with avelumab monotherapy, and an ORR of 17%, a PFS of 3.9 months and a median OS of 7.8 months in the combination arm [36]. These results suggest a promising activity of dual PD-L1 and EGFR blockade in SCAC, although notably the OS was longer in the group treated with avelumab alone.

The anti-PD-1 antibody retifanlimab was evaluated in the phase II POD1UM-202 trial in patients with locally advanced or metastatic SCAC who progressed after platinum-based chemotherapy, achieving an ORR of 13.8%, a median PFS of 2.3 months, and a median OS of 10.1 months [87]. These results supported the FDA approval of retifanlimab as a single agent in patients with locally recurrent or metastatic SCAC with disease progression on or intolerance to platinum-based chemotherapy [80].

Retifanlimab was also evaluated as a first-line treatment in the phase III POD1UM-303/InterAACT2 trial [88]. In this study, the addition of retifanlimab to carboplatin and paclitaxel in patients with locally recurrent or metastatic SCAC significantly improved clinical outcomes compared with chemotherapy alone, with a median PFS of 9.3 vs. 7.4 months and an OS of 32.8 vs. 22.2 months [88,89]. Therefore, retifanlimab was recently approved as first-line treatment in combination with chemotherapy by both the EMA and FDA.

By contrast, the phase II SCARCE-PRODIGE 60 study, which evaluated the addition of atezolizumab to mDCF as a first-line treatment for advanced SCAC, did not demonstrate an improvement in PFS and did not meet its primary endpoint, while reporting an increase in adverse events of grade 3 or higher [90]. Nevertheless, the exploratory analysis suggested a possible benefit in patients with PD-L1 CPS ≥ 5 [90].

Several novel immunotherapy-based strategies are currently being explored, including combinations of ICIs with anti-VEGF, anti-EGFR, and TGF-β-directed agents and with HPV-targeted therapeutic vaccines [80].

Overall, immunotherapy has become an increasingly important component of the treatment landscape for SCAC, particularly in the advanced setting. However, clinical benefit varies considerably from patient to patient, and, as discussed below, no biomarker has demonstrated sufficient analytical and clinical validity to guide immunotherapy selection in routine clinical practice, so far.

### 4.1. PD-L1

PD-L1 positivity has been reported in a substantial proportion of tumors, although with wide variations depending on the assay, antibody, scoring system and positivity threshold used: in different studies, PD-L1 positivity has been defined using either tumor proportion score (TPS) or CPS, with several thresholds including TPS ≥ 1%, CPS ≥ 1, CPS ≥ 5 or CPS > 40, thus limiting the comparability between different trials.

Several studies evaluated the prognostic role of PD-L1 expression, with conflicting results. While some reported an improvement in OS in patients with PD-L1 expression [91,92], others have found it to be a negative prognostic biomarker [93]. In addition, in localized or locally advanced disease, PD-L1 expression has been associated with better outcomes after CRT [77,94], but other studies have found no significant correlation [95].

In advanced or metastatic disease, PD-L1 expression has been associated with increased benefit from PD-1/PD-L1 inhibitors. In the KEYNOTE-158 trial, responses to pembrolizumab were higher in PD-L1-positive tumors (defined by CPS ≥ 1) than in PD-L1-negative tumors, with an objective response in 15% vs. 3% of patients, respectively [86]. Exploratory analyses from the SCARCE-PRODIGE 60 trial suggested a possible benefit from atezolizumab plus chemotherapy among patients with PD-L1 CPS ≥ 5 [90]. In addition, in the CARACAS trial, high PD-L1 expression (CPS > 40) was significantly more frequent in patients with disease control (stable disease or partial response), and was associated with longer OS and PFS [14].

In the POD1UM-202 trial, higher PD-L1 mRNA expression was associated with longer OS in patients treated with retifanlimab, although responses to treatment were observed regardless of PD-L1 expression [87].

Overall, although PD-L1 expression may be associated with better response to ICIs, it is not currently a sufficiently validated and reliable biomarker to select or exclude patients from immunotherapy.

### 4.2. TILs

Tumor-infiltrating lymphocytes (TILs) and, more in general, the inflammatory tumor microenvironment, appear to have a relevant prognostic and predictive value in SCAC. In HPV-related tumors, viral antigens may promote the recruitment of cytotoxic CD8+ TILs and activate antitumor immune responses, contributing to both CRT sensitivity and response to ICIs [96].

In localized disease, a high TILs infiltration has been consistently associated with more favorable outcomes after CRT: in two cohorts of patients with p16-positive tumors, relapse-free rates were markedly higher in patients with high levels of TILs compared to those with absent or low TILs (92% vs. 63%, respectively) [97]. Similarly, in a cohort of 150 patients treated with CRT, increases in CD8+ TILs and PD-1+ TILs were associated with improved local control and disease-free survival [77]. These findings support the concept that HPV-positive, TIL-rich SCAC is characterized by an immune-activated phenotype that confers sensitivity to treatment, and may also explain the delayed tumor regression frequently observed after CRT, as demonstrated by the ACT II trial, in which complete clinical response rates continued to increase from 52% at 11 weeks to 78% at 26 weeks after the start of treatment [96,98].

In the advanced/metastatic setting, the NCI9673 trial reported higher baseline levels of CD8+ TILs and granzyme B+ TILs in patients who responded to nivolumab treatment [83]. However, in a translational analysis of the CARACAS trial, high TIL infiltration was paradoxically associated with worse outcomes, possibly because TILs were assessed only on hematoxylin–eosin sections without IHC distinction between cytotoxic and regulatory subsets [14].

The prognostic and predictive relevance of the inflammatory tumor microenvironment is also being recognized in other squamous cell carcinomas. In esophageal squamous cell carcinoma (ESCC), for example, PSD3 is significantly upregulated and associated with poor prognosis, and has been linked to autophagy-associated immune modulation and regulation of MHC-I expression [99]. The inhibition of PSD3 may therefore enhance tumor immunogenicity, supporting its role as a potential therapeutic target that could also warrant investigation in the context of SCAC.

### 4.3. TMB

Tumor mutational burden (TMB) in SCAC is generally low, with reported median values of 2.5–5 somatic mutations per megabase (mut/Mb) on whole-exome sequencing [13,19]. This is consistent with findings in other HPV-related malignancies, such as cervical carcinoma and HNSCC [12]. Nevertheless, TMB-high status, defined as ≥10 mut/Mb, has been reported in approximately 17% of SCAC cases [13].

Results from the KEYNOTE-158 trial led to the FDA approval of pembrolizumab for TMB-high solid tumors in 2020 [100]. However, this study reported no significant improvement in ORR among TMB-high SCAC compared to non-TMB-high SCAC (7% vs. 11%, respectively) in contrast to what emerged for most of the other tumor types included in the trial, although median tissue TMB score was higher in patients with a response than in patients without a response [101]. Similarly, no association between TMB status and OS was observed in the POD1UM-202 trial in patients treated with retifanlimab [87]. By contrast, in the CARACAS trial, TMB-high status was significantly associated with an improved OS in patients treated with avelumab with or without cetuximab [14].

Overall, available data do not support the use of TMB-high status as a reliable predictive biomarker for immunotherapy response in SCAC.

### 4.4. MSI

Mismatch repair deficiency (MMRd) and microsatellite instability (MSI) are rare in SCAC.

Alterations in the key components of the DNA mismatch repair system (i.e., MLH1, MSH2, MSH6, and PMS2) lead to the progressive accumulation of mutations within microsatellites, which are repetitive DNA sequences dispersed throughout the genome, leading to the phenotype known as microsatellite instability (MSI) [102]. MMRd/MSI status emerged as a valid predictive biomarker for immunotherapy in several tumors, including CRC and G/GEJ adenocarcinomas [103,104,105].

In SCAC, MMRd tested by IHC has been described in 1% of cases [16], while a recent study reported MSI status tested by NGS in 1.7% of patients [13]. In the phase II KEYNOTE-158 trial, which demonstrated the clinical benefit of pembrolizumab in patients with advanced non-colorectal MMRd/MSI cancers, only one of the 233 evaluated patients had an anal carcinoma [106].

Notably, high TMB does not appear to be associated with MSI status in SCAC [13,107], in contrast to most other tumors, including CRC and G/GEJ adenocarcinomas, which show high concordance between TMB-high and MSI tumors [107].

Despite its low prevalence, MMRd/MSI status is relevant because of its robust predictive value for response to ICIs across multiple tumor types. However, MSI/MMR testing is currently not required for the adoption of immunotherapy in SCAC, and the available evidence is too limited to define its predictive value in this disease.

## 5. Future Perspectives

Despite the growing number of molecular alterations that have been identified, clinically validated biomarkers are still lacking. This highlights the gap that still exists between molecular characterization and the implementation of precision oncology in SCAC.

Future research should therefore focus on developing robust molecular classifications capable of identifying biologically distinct subgroups and guiding personalized therapeutic approaches. The increasing availability of comprehensive genomic, transcriptomic, and spatial profiling technologies will be helpful in better understanding tumor heterogeneity, tumor-immune interactions, and mechanisms of treatment resistance.

Liquid biopsy approaches, including ctDNA analysis, could be useful for the non-invasive assessment of disease burden, treatment response, early detection of recurrence, and identification of actionable alterations. In HPV-associated SCAC, pretreatment HPV ctDNA levels have generally been shown to correlate with disease burden and tumor stage, including lymph node involvement and metastatic disease [12,108,109]. Moreover, ctDNA levels generally decrease during CRT or systemic chemotherapy, and persistent ctDNA following treatment has been associated with worse outcomes [12,108,109,110,111]. Liquid biopsy can also complement tissue-based molecular profiling, allowing the identification of genomic alterations and potentially actionable targets especially when performing tissue biopsies is not feasible. In one series, liquid and tissue biopsies were highly concordant in identifying genomic alterations in SCAC, and molecular alterations detected through liquid biopsy allowed 8 of 44 patients to access additional treatments [13].

Additional therapeutic approaches and novel biomarkers are also being explored in squamous cell carcinomas of other sites. These include strategies targeting NF-κB, which have shown promising results in preclinical studies focusing on oral squamous cell carcinomas [112], and high membrane progesterone receptor α (mPRα) protein expression, which has been associated with poor OS in lung squamous cell carcinoma, supporting its potential role both as a prognostic biomarker and a therapeutic target [113]. The ongoing research for novel predictive and prognostic biomarkers and therapeutic targets across different squamous cell carcinomas could provide a rationale for future investigations in SCAC.

## 6. Conclusions

In recent years, our understanding of the molecular biology of SCAC has significantly expanded, revealing a much more complex and heterogeneous disease than previously thought, and highlighting several new potential opportunities for therapeutic intervention. Immunotherapy has emerged as an important component of the treatment landscape, particularly in advanced stages, and the combination of immunotherapy with molecularly targeted agents represents a promising strategy.

Ultimately, collaborative multicenter studies and molecularly driven clinical trials will be essential in order to validate emerging biomarkers and to identify patients most likely to benefit from specific therapies. Integrating molecular profiling into routine clinical practice has the potential to transform the management of SCAC, moving from a largely uniform treatment paradigm to a more personalized, biomarker-driven approach.

## Figures and Tables

**Table 1:** Main molecular alterations and potential targeted treatments in SCAC.

	Frequency of Alterations (%) *	Potential Targeted Treatments
**PI3K/AKT/mTOR pathway**		
*PIK3CA*	32%	PI3K, AKT and mTOR inhibitors
*PTEN*	13%	
*FBXW7*	12%	
*STK11*	5%	
*AKT1*	2%	
**Histone modification and chromatin remodeling**		
*KMT2D*	18%	
*KMT2C*	14%	
*EP300*	6%	
*ARID1A*	3%	
**Wnt/β-catenin pathway**		
*FAT1*	9%	
*APC*	3%	
*CTNNB1*	2%	
**MAPK pathway**		
*KRAS*	2%	RAS inhibitors
*HRAS*	1%	
*NRAS*	1%	
*BRAF*	1%	BRAF inhibitors
**Receptor tyrosine kinases**		
*FGFR3*	3%	FGFR inhibitors
*FGFR1*	1%	
*FGFR2*	1%	
*EGFR*	2%	EGFR inhibitors
*ERBB2*	2%	HER2-targeted agents
**DNA damage response**		
*ATM*	4%	PARP inhibitors
*BRCA1*	3%	
*BRCA2*	3%	
**Cell cycle regulation**		
*TP53*	12%	
*CDKN2A*	6%	
*RB1*	4%	
*TERT*	7%	
**Others**		
*CYLD*	10%	
*SOX2*	9%	
*NOTCH1*	6%	

* Frequencies obtained by aggregating data from the studies included in the analysis [10,13,14,15,16,17,18,19,20,21,22].

## Data Availability

The data analyzed in this study are available from the corresponding author upon reasonable request.
